# Prevalence of Subclinical Hypothyroidism in Pregnancy and Its Association With Anti-thyroperoxidase Antibody and the Occurrence of Gestational Diabetes Mellitus

**DOI:** 10.7759/cureus.21087

**Published:** 2022-01-10

**Authors:** Prakruti Dash, Rajlaxmi Tiwari, Saurav Nayak, Saubhagya K Jena, Manaswini Mangaraj

**Affiliations:** 1 Biochemistry, All India Institute of Medical Sciences, Bhubaneswar, Bhubaneswar, IND; 2 Biochemistry, Institute of Medical Sciences and SUM Hospital, Bhubaneswar, IND; 3 Obstetrics and Gynecology, All India Institute of Medical Sciences, Bhubaneswar, Bhubaneswar, IND

**Keywords:** prevalence, euthyroid, anti tpo ab, gdm, sch

## Abstract

Introduction

Subclinical hypothyroidism (SCH) and gestational diabetes mellitus (GDM) are common endocrinological abnormalities associated with pregnancy. The presence of a raised anti-thyroperoxidase (anti-TPO) antibody titer increases the risk of progression of subclinical hypothyroidism to overt hypothyroidism. Subclinical hypothyroidism and GDM are known to affect maternal and fetal outcomes adversely. A few studies have shown an increased risk of GDM with autoimmune hypothyroidism. However, data regarding this association between GDM, SCH, and anti-TPO Ab are scarce. This study aimed to find the prevalence of autoimmune subclinical hypothyroidism and its association with GDM in pregnancy.

Materials and methods

In a cross-sectional study, 382 pregnant women at their first antenatal checkup (ANC) were enrolled in the study. Serum thyroid-stimulating hormone (TSH), free T4 (FT4), anti-TPO Ab, and the 75 g oral glucose tolerance test (OGTT) were evaluated. The results obtained were analyzed in Systat Version 13.2 (SPSS Inc., Chicago, IL).

Observations

Results showed an SCH prevalence of 37.69% with a raised anti-TPO Ab titer in 49.31% of the diagnosed SCH cases, pointing towards an autoimmune etiology. Our study revealed a GDM prevalence of 12.04%. Out of the 46 GDM cases, 16 were found to have SCH and 3 cases had raised anti-TPO Ab titers. In our study, 27.73% of euthyroid pregnant women had a raised anti-TPO Ab titer. Our study revealed no significant association between GDM, SCH, and raised anti-TPO Ab titer.

Conclusion

Anti-TPO antibody subsequently leads to hypothyroxinemia, for which it is necessary that cases with high titer of anti-TPO antibody though euthyroid should be meticulously followed up and screened for to detect development of hypothyroidism or SCH, particularly in future pregnancies. However, GDM prevalence was at par with the national figure, but with no significant association of SCH and a high anti-TPO ab titer was found with GDM in our study. Further studies with a larger cohort may establish a causal association between the two most common endocrinological disorders observed in pregnancy.

## Introduction

Altered (hypo or hyper) thyroid gland functioning is one of the most common endocrinological abnormalities associated with pregnancy [1,2]. Thyroxine concentration is also related to deiodinase released by the placenta as well as the thyrotropic action of beta-human chorionic gonadotropin (HCG), whose concentration increases in pregnancy [3,4]. As the growing fetus and placenta depend upon maternal thyroid hormones in the first trimester for their growth and development, an imbalance in maternal thyroid status affects both, resulting in adverse obstetric outcomes [5]. Previous studies have documented abortion, abruptio placentae, preeclampsia, intrauterine growth retardation (IUGR), low birth weight, etc., is commonly observed in pregnant women with altered thyroid status [6-11].

Few long-term follow-up studies have depicted intellectual deficits in children born to mothers with hypothyroidism as well as hyperthyroidism, highlighting the role of maternal thyroid hormone status in the growth and development of children in the long run [12]. In several studies, repercussions of abnormal thyroid hormone status have also been noted on the villous and extra-villous trophoblastic proliferation, affecting the viability of the placenta [13,14].

The incidence of hypothyroidism in pregnancy is higher in Asian countries, with more observed in the Indian population being attributed to nutritional as well as immunological origins. Even subclinical hypothyroidism (SCH) with high thyroid-stimulating hormone (TSH) and a normal thyroxine level is commonly associated with endocrine abnormalities in pregnancy [15-19]. Anti-thyroperoxidase (anti-TPO) antibody having the ability to cross the placenta has been suggested to affect fetal growth [20,21]. Euthyroid pregnant women with high anti-TPO antibody titers have been registered with several adversities in obstetric and fetal outcomes [22-24].

Gestational diabetes mellitus (GDM) is a frequent occurrence in the second trimester of pregnancy, with the risk being greater with increasing age [25-27]. Autoimmune diseases like insulin-dependent diabetes mellitus (IDDM), Hashimoto’s thyroiditis, pernicious anemia, etc., are more common in women and occur concomitantly. An association between hypothyroidism and different types of diabetes mellitus has been reported previously [28-30].

Various studies have documented beta-cell dysfunction and insulin resistance as the basis of the development of GDM [31]. Inflammatory events associated with autoimmune disorders and insulin resistance are suggested to be the potential link between the two conditions. Insulin resistance was found to be associated with autoimmune thyroid dysfunction with a raised titer of anti-TPO antibodies, signifying a possible relationship between autoimmune thyroid dysfunction and GDM. Some studies have documented an increased risk of GDM with hypothyroidism and a raised titer of anti-TPO Ab [32-35], whereas some others have registered no significant association between GDM and autoimmune thyroid disorders in pregnancy [36,37]. Yang et al. in their meta-analysis showed that there was a significant but not strong association between thyroid antibodies and the risk of GDM [38].

Moreover, data regarding the prevalence of hypothyroidism and autoimmunity in women with GDM are scarce. Hence, the following study was conducted in a tertiary care center to estimate the prevalence of subclinical hypothyroidism in pregnant women, its association with raised anti-TPO antibody titer, and the occurrence of GDM detected at their first antenatal visit.

## Materials and methods

This cross-sectional study was conducted in the Department of Biochemistry, in collaboration with the Department of Obstetrics and Gynecology, and 382 eligible pregnant women coming for their first antenatal checkup (ANC) were enrolled in the study.

Apparently healthy pregnant women, both primigravida and multi-gravida, with singleton pregnancies in their first ANC were included and written informed consent was obtained from the enrolled cases. Pregnant women with preexisting thyroid diseases or any other endocrine disorders, pre-existing diabetes, on any hormone replacement therapy, any other metabolic or chronic disorders, and bad obstetric history with a known cause were excluded from the study.

After general and gynecological examination, fasting, one-hour, and two-hour blood samples were collected for 75 g OGTT and estimation of thyroid profile (TSH, fT4, anti-TPO antibody). The biochemical parameters were performed on the Beckman Coulter AU5A00 auto analyzer with commercially available kits. Thyroid profiles were done by the chemiluminescence method in an Siemens Advia Centaur automated Immunoassay analyzer.

For this study, the trimester-specific upper limit value for TSH was taken as <2.5 mIU/mL for the first trimester and <3 mIU/mL for the second and third trimesters as per American Thyroid Association (ATA) 2011 criteria. Patients with TSH levels higher than the trimester specific level and normal fT4 levels were diagnosed with SCH. Anti-TPO level <60 U/L was taken as normal upper limit as per manufacturer’s protocol. Level more than 60U/L is considered a raised anti-TPO titer.

GDM was diagnosed using 75 g of glucose challenge test (GCT) with a fasting value of more than 92 mg/dl, a one-hour post-glucose value of more than 180 mg/dl, and a two-hour post-glucose value of more than 153 mg/dl.

The study has got ethical clearance from the Institute Ethics Committee.

The results obtained were statistically analyzed with Systat Version 13.2 (SPSS Inc., Chicago, IL). The normality distribution of the data was assessed by the Shapiro-Wilk test. Data were presented as a median value. One-way analysis of variance (ANOVA) was put in to see the significance. A p-value of <0.05 was considered to be statistically significant.

## Results

**Figure 1:**
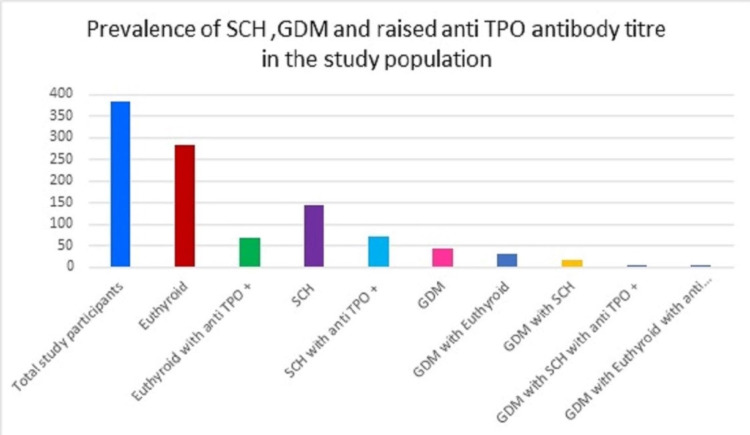
Prevalence of SCH, GDM, and raised anti-TPO antibody titer in the study population SCH: subclinical hypothyroidism, GDM: gestational diabetes mellitus, TPO: thyroperoxidase antibody

The number of pregnant women screened was 382 at a gestational age of between 11 and 34 weeks, with a median of 25 weeks. The prevalence of SCH in pregnancy in our study was found to be 37.69% (144 out of 382 study participants). The presence of a raised anti-TPO Ab titer was seen in 49.31% of SCH cases, indicating an autoimmune etiology. The prevalence of GDM was 12.04% (46 out of 382 study participants) in our study, and the number of cases associated with SCH was seen to be 34.7% (16 cases), and both SCH and raised anti-TPO Ab titer were observed in three cases (6.5%). Thirty cases (65.1%) of GDM were euthyroid and out of those, four (8.5%) cases were found to have raised anti-TPO Ab titer (Figure 1).

The serum TSH value was highest in cases with a raised anti-TPO Ab titer that was statistically significant (p<0.01). We observed that the data were not normally distributed as the normality test (Shapiro-Wilk) had a p-value of ≤0.001. As a result, all of the data are represented as median (IQR), and a one-way ANOVA was performed to determine significance (Table 1). 

**Table 1:** Median value and range of the study parameters TSH: thyroid stimulating hormone, SCH: subclinical hypothyroidism, ATPO: anti-thyroperoxidase, FT4: free thyroxine, GCT: glucose challenge test, FBS: fasting blood sugar, PGBS: post-glucose blood sugar

Sl. No.	Parameter	Controls (SCH absent N = 238 median (IQR))	SCH+/ATPO+N = 71 median (IQR)	SCH+/ATPO−N = 73 median (IQR)	One way ANOVA (p-value)
1	TSH mIU/mL	1.575 (1.19–1.992)	3.84 (3.075–4.605)	3.37 (2.95–4.5)	≤0.001*
2	FT4 ng/dL	1.145 (1–1.3)	1.1 (0.995–1.235)	1.17 (1.1–1.3)	0.076
3	Anti-TPO Ab U/L	44.9 (29.35–60.075)	110 (77.9–201.5)	33.5 (28–44)	≤0.001*
4	75 g GCT FBS in mg%	90 (84–117)	102 (85.5 120.5)	99 (65 – 106)	0.630
5	75 g GCT 1 hour PGBS in mg%	132 (100–165)	160 (110–200)	148 (110–172)	0.670
6	75 g GCT 2 hour PGBS in mg%	110 (98–124)	146 (100–189)	134 (100–186)	0.754

Table 2 represents the number of SCH cases showing a raised anti-TPO Ab titer (>60 U/L). Seventy-one out of 144 pregnant women with SCH in our study had a raised anti-TPO Ab titer in their serum. Similarly, 27.73% (66 out of 282) of the study participants with euthyroid status showed a raised anti-TPO Ab titer in their serum.

**Table 2:** : Anti-TPO antibody titer in the study population Anti-TPO: anti-thyroperoxidase, SCH: subclinical hypothyroidism, U/L: units/liter

Total n=382	Anti-TPO antibody titer >60 U/L	Anti TPO antibody titer <60 U/L
Euthyroid N=238	66 (27.73%)	172 (72.2%)
SCH N=144	71 (49.31%)	73 (50.69%)

We observed that GDM had a prevalence of 12.04% (46 cases out of 382 participants) in the study population. Out of the 46 cases, 16 had both GDM and SCH, and 3 of them were observed to have raised anti-TPO Ab along with SCH and GDM. Of the 30 euthyroid cases diagnosed with GDM, four participants had a raised anti-TPO Ab titer in their serum (Table 3).

**Table 3:** Prevalence of GDM with SCH and raised ATPO antibody titer in the study population GDM: gestational diabetes mellitus, SCH: subclinical hypothyroidism, ATPO: anti-thyroperoxidase antibody

Group	Anti-TPO titer >60U/L	Anti-TPO titer <60U/L
Total GDM cases n=46 (12.04%)		
GDM with SCH (n=16)	03 (6.5%)	13 (28.2%)
GDM with euthyroid (n=30)	4 (8.6%)	26 (56.5%)

In our study, we could not find any increased risk of GDM associated with SCH and raised anti-TPO Ab titer in the study population (Table 4).

**Table 4:** Relative risk of GDM compared with SCH with ATPO positive vs SCH with ATPO negative vs controls (no SCH) GDM: gestational diabetes mellitus, SCH: subclinical hypothyroidism, ATPO: anti-thyroperoxidase antibody, RR: relative risk

Sl. No.	Comparison	RR	p-Value
1	SCH with ATPO positive vs SCH with ATPO negative	0.996	1.000
2	SCH with ATPO positive vs euthyroid	1.010	0.836
3	SCH with ATPO negative vs euthyroid	1.006	0.839

## Discussion

This study was aimed at evaluating the prevalence of subclinical hypothyroidism during pregnancy in a tertiary-level hospital in Eastern India. The occurrence of raised anti-TPO Ab titer and association of GDM with SCH and raised anti-TPO Ab titer were also evaluated compared to euthyroid cases.

Globally, the prevalence of hypothyroidism in pregnant women is reported to be extremely variable [39]. In India, reports on the prevalence of hypothyroidism in pregnancy range from 1.2% to more than 60% in several studies. In a meta-analysis of 61 studies across 60,066 study subjects by Yadav et al., the prevalence of overt hypothyroidism in India was documented to be 11.07% [40].

The prevalence of SCH in pregnancy differs extensively worldwide [41]. In India, the prevalence of SCH varies from 2.8% to 32.94% in different parts of the country, as documented in various studies [42-45].

Gayathri et al. reported the prevalence of SCH of 2.8% among pregnant women in Chennai and 57.1% of the subclinical hypothyroid patients had positive TPO antibodies [42]. Aggarwal et al. documented the prevalence of SCH to be 10.9% among pregnant women in a study conducted in a premier institute in north India, and TPO antibody positivity was 59% among the subclinical hypothyroid pregnant women in their study [43]. Another study conducted in Delhi by Dhanwal et al. has cited an even higher prevalence of SCH (13.8%) among pregnant women and a good number (57%) of them were TPO antibody positive [44]. All the studies mentioned above used a cut-off value of TSH >4.5 μIU/ml to diagnose SCH.

In a study by Mandal et al., 32.94% of the pregnant mothers had subclinical hypothyroidism and anti-TPO Ab positivity was found in 12.15% of the study population and in 33.93% of the SCH pregnant women [45]. Thus, it is evident that the prevalence of SCH varies widely in different parts of the country among pregnant women.

In our study, we documented a comparatively higher prevalence of subclinical hypothyroidism, i.e., 37.69%, which is quite high compared to other studies but almost in accordance with Mandal et al. as we used ATA 2011 criteria with an upper limit of TSH of 2.5 mIU/L in the first trimester and 3 mIU/L in the second and third trimesters.

In the overall study population of our study, 49.31% of SCH cases were found to have anti-TPO positivity in their serum. Compared to studies done by Gayathri et al., Aggarwal et al., and Dhanwal et al. [42-44], the percentage of SCH cases having anti-TPO antibody positivity was lower in our study population. This may be attributed to iodine deficiency as the most common cause of lower levels of thyroid hormones and auto immunity coming next to it in our geographical distribution.

The percentage of euthyroid subjects showing raised anti-TPO Ab titers was high in our study. The high prevalence of SCH in pregnancies in our study and the raised level of anti-TPO antibody titer observed in euthyroid subjects may give an alarm and reinforce the need for regular and mandatory screening of thyroid hormone status in pregnancy along with screening for anti-TPO Ab. Women with euthyroid status but with raised anti-TPO Ab positivity should ideally be followed up for more rigorous assessment of hypothyroidism in subsequent pregnancies.

The prevalence of GDM in our study was found to be 12.04%. GDM coexisted with SCH in 16 study participants. Out of the 16 cases of GDM with SCH, 3 cases were observed to have raised anti-TPO titers. Similarly, among the 30 GDM cases with euthyroid status, 4 cases were found to have raised anti-TPO Ab titers.

The prevalence of GDM in India, as per current statistics, varies from 4% to 18%. Literature also shows the prevalence rate is higher in urban areas than in rural areas. Our study population was heterogenous in geographical and social distribution. We observed a prevalence rate of 12.04%, which is in accordance with the documented data [25,26].

Many authors [46, 47] have reported the occurrence of hypothyroidism and raised anti-TPO Ab titers amongst women with GDM. Many studies have documented an increased risk of GDM in hypothyroidism associated with high anti-TPO Ab titers during pregnancy [48-51]. In our study, however, we found no statistically significant association between GDM, SCH, and high anti-TPO antibody titer.

Relative risk assessment of GDM in association with SCH and raised anti-TPO Ab titer versus SCH with normal anti-TPO Ab titer versus euthyroid revealed no statistically significant value.

## Conclusions

The present study revealed an increase in subclinical hypothyroidism in pregnancy in our geographical area. A significant number of SCH with high anti-TPO antibody titer points towards autoimmunity as being a significant cause of the decreased level of thyroid hormones in pregnancy. However, GDM prevalence was at par with the national figure but with no significant association of SCH, and a high anti-TPO ab titer was found with GDM in our study.

Further longitudinal studies with a larger cohort may establish a causal association between the two most common endocrine disorders observed in pregnancy. Looking at the adverse impact of hypothyroidism on pregnancy outcomes as registered by various studies, mandatory screening for thyroid status in pregnancy has been approved by various regulatory bodies and is now in practice. However, screening for autoimmunity with measurement of anti-TPO ab titer is currently not done regularly. As anti-TPO antibody subsequently leads to hypothyroxinemia as per documented reports, it is necessary that cases with a high titer of anti-TPO antibody though euthyroid particularly in primigravida cases should be meticulously followed up and screened for to detect the development of hypothyroidism or SCH, particularly in future pregnancies. Undiagnosed thyroid dysfunction and autoimmunity might potentially amplify the maternal and fetal complications associated with hypothyroxinemia.
